# Supplementary material for: Somatosensory neurons integrate the geometry of skin deformation and mechanotransduction channels to shape touch sensing
Source: eLife. 2019 Aug 13;8:e43226. doi: 10.7554/eLife.43226 (PMC6692131; doi:10.7554/eLife.43226)
Supplement: Transparent reporting form [file elife-43226-transrepform.docx]

***eLife’s* transparent reporting form**

We encourage authors to provide detailed information *within their submission* to facilitate the interpretation and replication of experiments. Authors can upload supporting documentation to indicate the use of appropriate reporting guidelines for health-related research (see [EQUATOR Network](http://www.equator-network.org/%20)), life science research (see the [BioSharing Information Resource](https://biosharing.org/)), or the [ARRIVE guidelines](http://www.plosbiology.org/article/info:doi/10.1371/journal.pbio.1000412) for reporting work involving animal research. Where applicable, authors should refer to any relevant reporting standards documents in this form.

If you have any questions, please consult our Journal Policies and/or contact us: [editorial@elifesciences.org](mailto:editorial@elifesciences.org).

**Sample-size estimation**

- You should state whether an appropriate sample size was computed when the study was being designed
- You should state the statistical method of sample size computation and any required assumptions
- If no explicit power analysis was used, you should describe how you decided what sample (replicate) size (number) to use

Please outline where this information can be found within the submission (e.g., sections or figure legends), or explain why this information doesn’t apply to your submission:

This study does not include any comparison of experimental results between groups, but rather focuses on using computation to decipher biophysical mechanisms. Consequently, no explicit power analysis was used. We aimed for n = 6-9 biological replicates (electrical recordings of identified neurons), similar to previous studies, but collected more to account for recordings that might not pass quality control. Measurement of cuticle deformation included n=2 biological replicates (adult animals). The number of replicates is indicated in the figure legends (Figure 2, Figure 4, Figure 5) and in Materials and Methods.

**Replicates**

- You should report how often each experiment was performed
- You should include a definition of biological versus technical replication
- The data obtained should be provided and sufficient information should be provided to indicate the number of independent biological and/or technical replicates
- If you encountered any outliers, you should describe how these were handled
- Criteria for exclusion/inclusion of data should be clearly stated
- High-throughput sequence data should be uploaded before submission, with a private link for reviewers provided (these are available from both GEO and ArrayExpress)

Please outline where this information can be found within the submission (e.g., sections or figure legends), or explain why this information doesn’t apply to your submission:

For the pre-indentation experiments, we used n = 11 recordings in separate worms (biological replicates), with n = 3-11 presentations of each stimulus (technical replicates) per recording. Both pre-indented and non-pre-indented data were collected for the same recordings, but not every recording included every displacement, so the number of biological replicates per displacement point varied from n = 5 to n = 11. Recordings were only included if they met the criteria outlined in the Data Analysis section of the experimental methods. Representative traces shown in Figure 4A are from one biological replicate.

**Statistical reporting**

- Statistical analysis methods should be described and justified
- Raw data should be presented in figures whenever informative to do so (typically when N per group is less than 10)
- For each experiment, you should identify the statistical tests used, exact values of N, definitions of center, methods of multiple test correction, and dispersion and precision measures (e.g., mean, median, SD, SEM, confidence intervals; and, for the major substantive results, a measure of effect size (e.g., Pearson's r, Cohen's d)
- Report exact p-values wherever possible alongside the summary statistics and 95% confidence intervals. These should be reported for all key questions and not only when the p-value is less than 0.05.

Please outline where this information can be found within the submission (e.g., sections or figure legends), or explain why this information doesn’t apply to your submission:

This study does not make use of summary statistics, but does report average values. For experimental data, average values are reported as mean and error bars as SEM (Figure 4, Figure 5D).

(For large datasets, or papers with a very large number of statistical tests, you may upload a single table file with tests, Ns, etc., with reference to sections in the manuscript.)

**Group allocation**

- Indicate how samples were allocated into experimental groups (in the case of clinical studies, please specify allocation to treatment method); if randomization was used, please also state if restricted randomization was applied
- Indicate if masking was used during group allocation, data collection and/or data analysis

Please outline where this information can be found within the submission (e.g., sections or figure legends), or explain why this information doesn’t apply to your submission:

Both pre-indented and non-pre-indented protocols were applied to the same recordings. No masking was used in data collection or analysis.

**Additional data files (“source data”)**

- We encourage you to upload relevant additional data files, such as numerical data that are represented as a graph in a figure, or as a summary table
- Where provided, these should be in the most useful format, and they can be uploaded as “Source data” files linked to a main figure or table
- Include model definition files including the full list of parameters used
- Include code used for data analysis (e.g., R, MatLab)
- Avoid stating that data files are “available upon request”

Please indicate the figures or tables for which source data files have been provided:

Source data are provided for experimental data that is reported here for the first time:

Figure 2B; Figure 4B, 4C, 4D; Figure 5D. The remaining experimental data were collected and reported previous in Ref. 19: Eastwood et al, PNAS, 2015.
